# The *Pid* Family Has Been Diverged into *Xian* and *Geng* Type Resistance Genes against Rice Blast Disease

**DOI:** 10.3390/genes13050891

**Published:** 2022-05-17

**Authors:** Ruipeng Chai, Jinyan Wang, Xing Wang, Jianqiang Wen, Zhijian Liang, Xuemei Ye, Yaling Zhang, Yongxiang Yao, Jianfu Zhang, Yihua Zhang, Ling Wang, Qinghua Pan

**Affiliations:** 1 Rice Blast Research Center, South China Agricultural University, Guangzhou 510642, China; cenivio@163.com (R.C.); oryza@stu.scau.edu.cn (J.W.); wangxing8714@126.com (X.W.); wenjianqiang51@126.com (J.W.); richardlzj@126.com (Z.L.); yexuemei316@126.com (X.Y.); byndzyl@163.com (Y.Z.); yaoyongxiang0415@163.com (Y.Y.); wangl@scau.edu.cn (L.W.); 2 College of Agronomy, Heilongjiang Bayi Agricultural University, Daqing 163319, China; 3 Dandong Academy of Agricultural Sciences, Dandong 118109, China; 4 Rice Research Institute, Fujian Academy of Agricultural Sciences, Fuzhou 350003, China; jianfzhang@163.com (J.Z.); zhangyihua80@163.com (Y.Z.)

**Keywords:** *Oryza sativa*, *Magnaporthe oryzae*, *Xian* and *Geng* type resistance gene, resistance function confirmation

## Abstract

Rice blast (the causative agent the fungus *Magnaporthe oryzae*) represents a major constraint on the productivity of one of the world’s most important staple food crops. Genes encoding resistance have been identified in both the *Xian* and *Geng* subspecies genepools, and combining these within new cultivars represents a rational means of combating the pathogen. In this research, deeper allele mining was carried out on *Pid2*, *Pid3*, and *Pid4* via each comprehensive FNP marker set in three panels consisting of 70 *Xian* and 58 *Geng* cultivars. Within *Pid2*, three functional and one non-functional alleles were identified; the former were only identified in *Xian* type entries. At *Pid3*, four functional and one non-functional alleles were identified; once again, all of the former were present in *Xian* type entries. However, the pattern of variation at *Pid4* was rather different: here, the five functional alleles uncovered were dispersed across the *Geng* type germplasm. Among all the twelve candidate functional alleles, both *Pid2*-ZS and *Pid3*-ZS were predominant. Furthermore, the resistance functions of both *Pid2*-ZS and *Pid3*-ZS were assured by transformation test. Profiting from the merits of three comprehensive FNP marker sets, the study has validated all three members of the *Pid* family as having been strictly diverged into *Xian* and *Geng* subspecies: *Pid2* and *Pid3* were defined as *Xian* type resistance genes, and *Pid4* as *Geng* type. Rather limited genotypes of the *Pid* family have been effective in both *Xian* and *Geng* rice groups, of which *Pid2*-ZS_*Pid3*-ZS has been central to the Chinese rice population.

## 1. Introduction

Rice (*Oryza sativa* L.), a crop domesticated in Asia and now cultivated worldwide, is used as a staple food for half of mankind [1,2,3,4,5]. As a result of two major and independent domestication events two different subspecies have been recognized, namely, ssp. *Xian* (*indica*) and ssp. *Geng* (*japonica*) [1,2,4,6]. The two genepools have differentiated over time through their been grown in distinct eco-geographical environments, and have diverged with respect to both the structure of the genome and their gene content [1,2,3,4,7]. Introgression from one gene pool to the other is seen as a useful strategy for increasing the crop’s genetic diversity.

One of the major pathogens of the rice crop is the fungus *Magnaporthe oryzae* Couch (syn. *Pyricularia oryzae* Cavara), the causative agent of the damaging disease rice blast [8,9,10,11,12,13]. A wealth of genes determining resistance to this pathogen has supported the success of using breeding to provide a sustainable means of mitigating the damage caused by blast [10,11,14,15,16]. The genetics of resistance largely follow the gene-for-gene principle, involving an interaction between a host’s resistance gene and a matching avirulence gene in the pathogen [17,18,19,20]. As a result, following the mutation of matching avirulence genes, major gene-based resistances are prone to rapid breakdown. This in turn enables the creation of a new genotype of resistance gene to overcome the emerged new race with its new resistance specificity [10,11,12,13,19,20,21,22]. New resistance specificities can be generated by mutations to a resistance gene’s coding sequence (CDS) as well as its regulating region, either in the form of single nucleotide polymorphic (SNP) or even multiple-nucleotide polymorphic mutations (called insertion/deletion, or InDel) [12,15,23,24,25,26,27,28]; where such changes result in an altered reaction to the pathogen, the mutation is referred to as a functional nucleotide polymorphism (FNP) [15,27,28]. As per the gene-for-gene principal, it is envisaged that a stronger arms race will lead to more FNPs emerging in any particular resistance gene [10,11,12,13,19,20,22]. Searching for FNPs in established host cultivars is considered an efficient way of identifying the novel resistances required for crop improvement [29,30,31,32,33].

At least 100 major genes encoding resistance to *M. oryzae* are known, an increasing number of which have been isolated [9,12]. Among the latter are the three genes *Pid2*, *-3*, and *-4*, present as a cluster on chromosome 6 (here after the *Pid* family), of which *Pid2* encodes for a B-lectin receptor kinase and the other two for nucleotide-binding site (NBS) and leucine-rich repeat (LRR) proteins [34,35,36]. The objective of the present study was to devise a set of reliable FNP markers based on variations in genomic sequences of the *Pid* family and to use these to exploit the extent of allelic variation available in rice germplasm. A particular focus was to reveal the genetic basis underlying resistance gene divergence between *Xian* and *Geng* subspecies.

## 2. Materials and Methods

### 2.1. Development of a Comprehensive FNP Marker System

The DNA sequences of *Pid2* (FJ915121.1), *Pid3* (FJ745364.1), and *Pid4* (MG839283.1) present in Digu as well as in a number of reference cultivars were retrieved from GenBank (http://www.ncbi.nlm.nih.gov/, accessed on 12 September 2020), and each set of alleles was aligned using Multalin (http://multalin.toulouse.inra.fr/multalin/, accessed on 13 September 2020). A comprehensive FNP marker system consisting of two sets of FNP markers, one for functional/non-functional haplotypes and another for individual alleles, was developed for deeper allele mining of each member of the *Pid* family. An interval sequence of each candidate FNP was subjected to various marker designations, including CAPS (cleaved amplified polymorphism sequences), and dCAPS (derived CAPS), using dCAPS Finder 2.0 (http://helix.wustl.edu/dcaps/dcaps.html, accessed on 28 September 2020). The necessary primer sequences were generated using Primer3 software (https://primer3.ut.ee, accessed on 28 September 2020). For sequencing largely diverged intervals, PCR was driven by triple and/or degenerate primers (Table S1) [37,38].

### 2.2. Marker Verification

The FNP assays were validated by testing a larger set of control cvs (called CKs), namely, Digu (DIG), Tetep (TTP), CO39, Zhenshan 97 (ZS97), Tadukan (TDK), Nipponbare (NPB), Koshihikari (KSH), and Shennong 265 (SN265). Each 20 µL PCR mixture contained 0.1 µL 5 U/µL rTaqase (TaKaRa, Dalian, China), 2.0 µL 10× rTaq Mg^2+^ plus buffer, 0.5 µL 10 mM dNTP (TaKaRa), 1.0 µL 2.5 µM primers (Sangon Biotech, Guangzhou, China), 1.0 µL 100 ng/µL template DNA, and 14.4 µL ddH_2_O. The PCR regime was initiated with a denaturing step (94 °C/3 min), which was followed by 35 cycles of 94 °C/30 s, 50–62 °C/30 s, 72 °C/25–30 s and completed with a 72 °C/5 min final extension. The resulting amplicons were digested for 3 h with the appropriate restriction enzyme (NEB Inc., Ipswich, MA, USA) at the recommended temperature in a 10 µL reaction containing 1.5 µL PCR product, 0.2 µL 3 U/µL enzyme, 1.0 µL 10× digestion buffer, and 8.3 µL ddH_2_O. The digested amplicons were electrophoretically separated through 10–12% polyacrylamide gels in the presence of Tris-boric acid–EDTA buffer and run at 250 V for 20–50 min, depending on the sizes of the PCR products.

### 2.3. Genotyping and Data Analysis

A smaller set of control cultivars, i.e., DIG, TTP, ZS97, NPB, SN265, and CO39, were involved in each genotyping experiment (Table S2). The functional and nonfunctional haplotypes of each member of the *Pid* family were first determined with two haplotype-specific FNP markers, then candidate functional alleles were determined with a set of allele-specific FNP markers by testing a regular panel consisting of 30 representative *Xian* and 30 *Geng* type cultivars. To confirm the genetic divergence of alleles between *Xian* and *Geng* rice groups (if any), allele mining was then extended to two additional germplasm panels, one consisting of 40 *Xian* type cultivars used as parents in rice breeding programs based in the southern province of Guangdong and the other of 28 *Geng* type cultivars used similarly in the north-eastern province of Heilongjiang (Table S2). An *χ*^2^ test was used to determine whether the two genepools had or had not experienced divergence. The test was based on the formula $\chi^{2}=\frac{N{\left[\left|\mathrm{ad}-\mathrm{bc}\right|-\left(\frac{1}{2}\right)N\right]}^{2}}{\left(a+b)(c+d)(a+c)(b+d\right)}$, where *a* and *b* represent the number of *Xian* type entries scored as respectively harbouring or not harbouring a given allele or genotype, while *c* and *d* represent the same for the *Geng* type entries. *N* denotes the total number of alleles or genotypes detected for each *Pid* gene or genotype [11,13,39]. If all alleles derived from a given resistance gene, which was extremely diverged into Xian group, then the resistance gene was defined as Xian type one, and that in turn called as Geng type one.

### 2.4. Validation of Candidate Functional Allele

The full length of each genomic sequence of two paired alleles, *Pid2*-ZS vs. *Pid2*-DIG and *Pid3*-ZS vs. *Pid3*-DIG, was amplified with Q5^®^ High-Fidelity 2X Master Mix (NEB Inc., Ipswich, MA, USA) and fused within a pGEM^®^-T Easy Vector (Promega Inc., Madison, WI, USA). Then, the correct fragment was digested with the common restriction enzyme *Asc* I and fused into the binary vectors pYLTAC380H [40] to form a construct carrying an individual allele. Each construct was transformed into the blast-susceptible cv. Nipponbare following Hiei et al. [41]. Phenotypes of the T_1_ transgenic plants were determined via challenge with the recipient cultivar-virulent isolate CHL346, and then the viable plants were further inoculated with the reference allele-avirulant isolates (ZB15 for *Pid2*-DIG [34] and Zhong-10-8-14 for *Pid3*-DIG [36]) according to Pan et al. [42]. The transgenic plants were confirmed by PCR-based genotyping with a set of three vector-related markers, namely, the selective marker (HYG) plus two directional vector-gene across markers (Table S1).

## 3. Results

### 3.1. Pid2 Alleles

**Figure 1:**
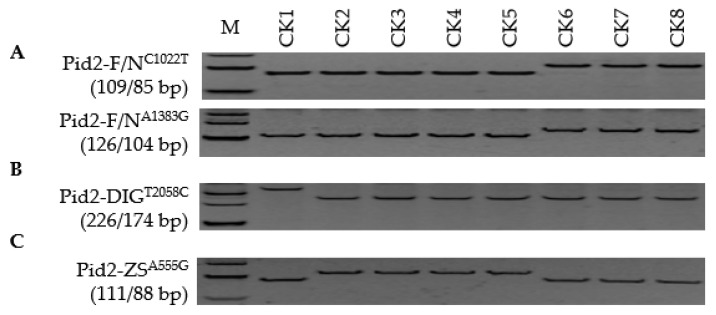
Development of a comprehensive FNP marker system able to distinguish both haplotypes and alleles of *Pid2*: (**A**–**C**) Discriminating between functional (F) and non-functional (N) haplotypes and between *Pid2*-DIG and *Pid2*-ZS alleles. CK1, Digu (*Pid2*-DIG); CK2, Tetep (*Pid2*-ZS); CK3, CO39 (*Pid2*-ZS); CK4, Zhenshan 97 (*Pid2*-ZS); CK5, Tadukan (*Pid2*-ZS); CK6, Nipponbare (*Pid2*-Null); CK7, Koshihikari (*Pid2*-Null); CK8, Shennong 265 (*Pid2*-Null). C1022T in superscript represents the FNP at the 1022 position of *Pid2*, C represents target, T non-target, and M the DL-500 size marker.

**Figure 2:**
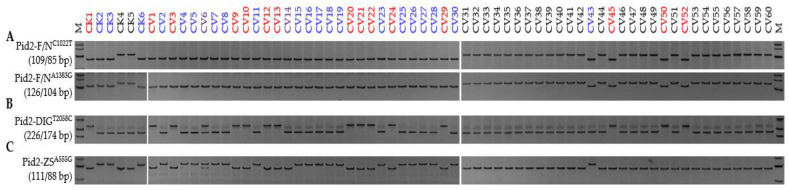
Alleles of *Pid2* represented in the regular panel consisting of both *Xian* (CV1-30) and *Geng* (CV31-60) types: (**A**) Functional and non-functional haplotypes; (**B**) *Pid2*-DIG allele; and (**C**) the *Pid2*-ZS allele. CK1, Digu (*Pid2*-DIG; red); CK2, Tetep (*Pid2*-ZS; blue); CK3, Zhenshan 97 (*Pid2*-ZS; blue); CK4, Nipponbare (*Pid2*-Null; black); CK5, Shennong 265 (*Pid2*-Null; black); CK6, CO39 (*Pid2*-ZS; blue); the undefined alleles carried by CV6 and CV14 are marked in purple. Detailed information on each entry is shown in Table S2. M represents the DL-500 size marker.

An alignment of *Pid2* CDSs of the fifteen reference cultivars revealed the presence of seven SNPs (Figure S1). A pair of FNPs, Pid2-F/N^C1022T^ and Pid2-F/N^A1383G^, effectively distinguished between the functional and the non-functional alleles. DIG, TTP, CO39, ZS97, and TDK each carried a functional allele, while NPB, KSH, and SN265 carried a non-functional one (Figure 1). Pid2-DIG^T2058C^ was informative for *Pid2*-DIG alleles (DIG) and Pid2-ZS^A555G^ for *Pid2*-ZS alleles (TTP, CO39, ZS97, and TDK). When the regular panel was screened, all 30 *Xian* type entries were found to carry a functional *Pid2* allele, while this was the case for only four of the 30 *Geng* types (Figure 2; Table S2). Of the 34 *Pid2* carriers, 14 belonged to *Pid2*-DIG and 18 to *Pid2*-ZS, while two carried a distinct allele (hereafter referred to as *Pid2*-New). The screen of the additional 40 *Xian* rice panel revealed that of the 39 carrying a functional copy of *Pid2*, 32 had the *Pid2*-ZS allele, six the *Pid2*-DIG allele, and one the *Pid2*-New allele. None of the additional *Geng* germplasm panel carried a functional copy of *Pid2* (Figure S2). A homogeneity test suggested that divergence of *Pid2* was specific to the *Xian* genepool (Table 1). It was, therefore, defined as a *Xian* type resistance gene.

### 3.2. Pid3 Alleles

**Figure 3:**
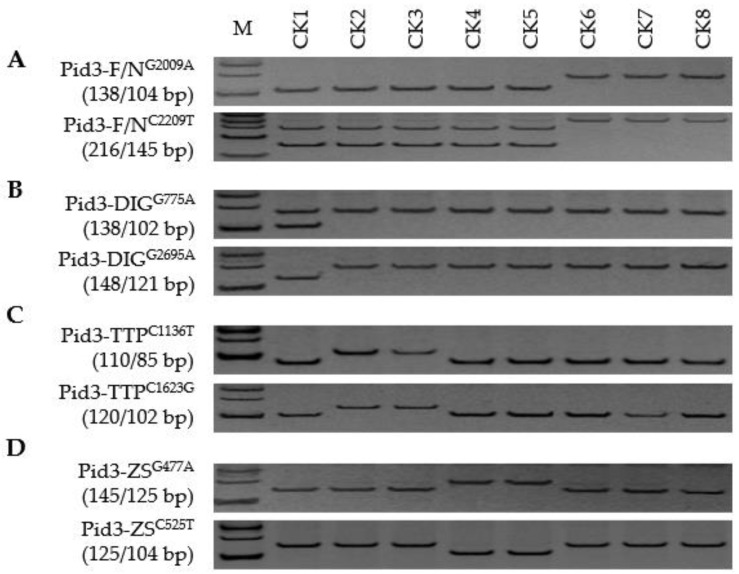
Development of a comprehensive FNP marker system able to distinguish both haplotypes and alleles of *Pid3*: (**A**–**D**) Discriminating between functional (F) and non-functional (N) haplotypes and among *Pid3*-DIG, *Pid3*-TTP, and *Pid3*-ZS alleles. CK1, Digu (*Pid3*-DIG); CK2, Tetep (*Pid3*-TTP); CK3, Tadukan (*Pid3*-TTP); CK4, Zhenshan 97 (*Pid3*-ZS); CK5, CO39 (*Pid3*-ZS); CK6, Nipponbare (*Pid3*-Null); CK7, Shennong 265 (*Pid3*-Null); CK8, Koshihikari (*Pid3*-Null). M represents the DL-500 size marker.

**Figure 4:**
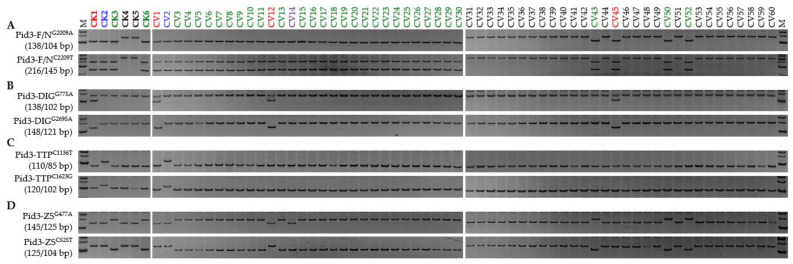
Alleles of *Pid3* represented in the regular panel consisting of both *Xian* (CV1-30) and *Geng* (CV31-60) types: (**A**) Functional and non-functional haplotypes; (**B**) the *Pid3*-DIG allele; (**C**) the *Pid3*-TTP allele; and (**D**) the *Pid3*-ZS allele. CK1, Digu (*Pid3*-DIG; red); CK2, Tetep (*Pid3*-TTP; blue); CK3, Zhenshan 97 (*Pid3*-ZS; green); CK4, Nipponbare (*Pid3*-Null; black); CK5, Shennong 265 (*Pid3*-Null; black); CK6, CO39 (*Pid3*-ZS; green); the undefined allele carried by CV14 is marked in purple. Detailed information on each entry is shown in Table S2. M represents the DL-500 size marker.

The variation in the *Pid3* CDSs identified in the fifteen reference cultivars comprised 29 SNPs and a single InDel (Figure S3); 18 of the SNPs and the InDel were targeted for marker development (data not shown). The Pid3-F/N^G2009A^ and Pid3-F/N^C2209T^ were both effective for distinguishing between functional and non-functional alleles: the five cultivars DIG, TTP, CO39, ZS97, and TDK each carried a functional allele, while NPB, KSH, and SN265 each carried a non-functional one (Figure 3; Table S2). Three pairs, Pid3-DIG^G775A^ vs. Pid3-DIG^G2695A^, Pid3-TTP^C1136T^ vs. Pid3-TTP^C1623G^, and Pid3-ZS^G477A^ vs. Pid3-ZS^C525T^, were confirmed as allele-specific FNP markers responsible for *Pid3*-DIG, *Pid3*-TTP, and *Pid3*-ZS, respectively (Figure 3). As was the case for *Pid2*, all 30 members of the *Xian* panel carried a functional *Pid3* haplotype, whereas only four of the *Geng* panel did (Figure 4; Table S2). The distribution of effective alleles was highly uneven: 29 of the *Pid3* carriers harboured the *Pid3*-ZS allele, three the *Pid3*-DIG allele, one the *Pid3*-TTP and one a novel allele (*Pid3*-New). The distribution was similarly uneven in the additional *Xian* panel, where 28 of the *Pid3*-positive entries carried the *Pid3*-ZS allele, three the *Pid3*-DIG allele, one the *Pid3*-TTP allele and one *Pid3*-New; none of the members of the additional *Geng* panel carried an effective allele (Figure S4). A homogeneity test implied that divergence at *Pid3* has occurred in the *Xian* genepool (Table 1); thus, it was termed a *Xian* type resistance gene.

### 3.3. Pid4 Alleles

**Figure 5:**
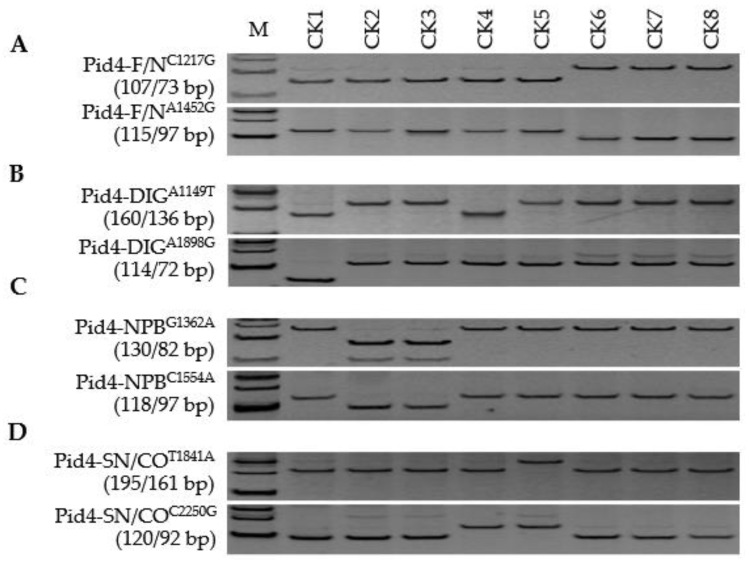
Development of a comprehensive FNP marker system able to distinguish both haplotypes and alleles of *Pid4*: (**A**–**D**) Discriminating between functional (F) and non-functional (N) haplotypes and among *Pid4*-DIG, *Pid4*-NPB, *Pid4*-CO, and *Pid4*-SN alleles. CK1, Digu (*Pid4*-DIG); CK2, Nipponbare (*Pid4*-NPB); CK3, Koshihikari (*Pid4*-NPB); CK4, CO39 (*Pid4*-CO); CK5, Shennong 265 (*Pid4*-SN); CK6, Tadukan (*Pid4*-Null); CK7, Tetep (*Pid4*-Null); CK8, Zhenshan 97 (*Pid4*-Null). M represents the DL-500 size marker.

**Figure 6:**
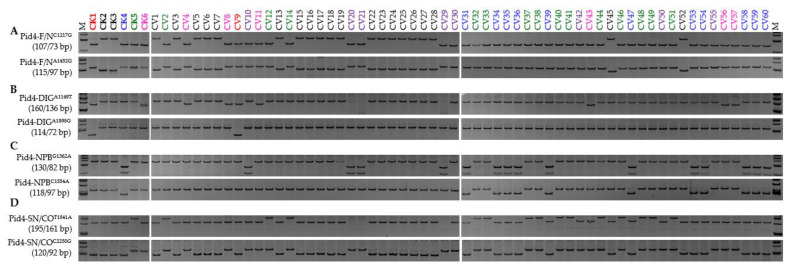
Alleles of *Pid4* represented in the regular panel consisting of both *Xian* (CV1-30) and *Geng* (CV31-60) types: (**A**) Functional and non-functional haplotypes; (**B**) the *Pid4*-DIG allele; (**C**) the *Pid4*-NPB allele; and (**D**) the *Pid4*-SN and *Pid4*-CO alleles. CK1, Digu (*Pid4*-DIG; red); CK2, Tetep (*Pid4*-Null; black); CK3, ZS97 (*Pid4*-Null; black); CK4, Nipponbare (*Pid4*-NPB; blue); CK5, Shennong 265 (*Pid4*-SN; green); CK6, CO39 (*Pid4*-CO; rose-red); the undefined alleles carried by CVs 10, 20, 21, 29, 30, 42, 50, and 55, are marked in purple. Detailed information on each entry is shown in Table S2. M represents the DL-500 size marker.

*Pid4* was by far the most diverse of the three members, with 149 SNPs and six InDels identified in the CDSs plus one intron of the thirteen reference cultivars (Figure S5); a sample of these (seventeen SNPs and two InDels) were targeted for marker development (data not shown). Both Pid4-F/N^C1217G^ and Pid4-F/N^A1452G^ were informative with respect to functionality: five cultivars, DIG, NPB, KSH, CO39, and SN265, were recognized as carriers of functional alleles, while TDK, TTP, and ZS97 harboured non-functional alleles (Figure 5; Table S2). Two pairs, Pid4-DIG^A1149T^ vs. Pid4-DIG^A1898G^ and Pid4-NPB^G1362A^ vs. Pid4-NPB^C1554A^, were confirmed as allele-specific FNP markers responsible for *Pid4*-DIG and *Pid4*-NPB, respectively, and Pid4-SN/CO^T1841A^ coupled with Pid4-SN/CO^C2250G^ as responsible for both *Pid4*-SN and *Pid4*-CO (Figure 5). Unlike the situation in *Pid2* and *Pid3*, functional *Pid4* alleles were present in many (28/30) of the *Geng* type entries, while the frequency of functional alleles was only moderate (12/30) in the *Xian* germplasm (Figure 6; Table S2). The distribution of the various alleles was even more than was the case for *Pid2* and *Pid3*, with fourteen entries carrying the *Pid4*-SN allele, eleven the *Pid4*-NPB allele, eight *Pid4*-New, and six the *Pid4*-CO allele (Figure 6). Extending the screen to the two additional panels revealed that 28/40 *Geng* type cultivars harboured a functional allele, while only 7/40 *Xian* type cultivars did. Of the 35 functional haplotypes, twelve were present in entries carrying the *Pid4*-CO allele, eleven in those carrying the *Pid4*-NPB allele, seven in those carrying *Pid4*-New, and five in those carrying the *Pid4*-SN allele, while a single entry carried the *Pid4*-DIG allele (Figure S6). A homogeneity test confirmed that significant divergence at *Pid4* has occurred in the *Geng* genepool (Table 1). Thus, it was termed a *Geng* type resistance gene.

### 3.4. Performance of Candidate Functional Allele

The transgenic T_1_ plants derived from the two paired alleles *Pid2*-ZS vs. *Pid2*-DIG and *Pid3*-ZS vs. *Pid3*-DIG revealed that both new functional alleles (*Pid2*-ZS and *Pid3*-ZS) expressed slightly higher resistance than their reference alleles (*Pid2*-DIG and *Pid3*-DIG; Figure S7). It has thus been demonstrated that the candidate functional alleles being explored by the comprehensive FNP marker systems are promising ones for conveying resistance.

## 4. Discussion

### 4.1. The Comprehensive FNP Marker Systems Have Largely Improved the Marker Works

In the present study, analysis via comprehensive FNP marker systems consisting of two set of FNP markers was carried out on the *Pid* family in the three panels consisting of 70 *Xian* and 58 *Geng* cultivars selected from various regions across the landrace and modern rice eras (Table 1 and Table S2). Each comprehensive FNP marker system for deeper allele mining of the *Pid* family was devised based on several criteria, including representative FNPs over a particular CDS, clear genotyping, and easy access for users [37,38]. As almost all resistance genes have diverged into functional and nonfunctional haplotypes (Figures S1, S3 and S5) [12,15,23,24,25,26,27,28,37,43], allele mining of each member of the *Pid* family was initiated with haplotype differentiation using a set of haplotype-specific FNP markers, enabling us to identify any new allele in each cultivar belonging to the functional haplotype. This means that allele mining could be stopped when there was not any functional haplotype in the panel (Figures S2 and S4). Then allele mining was pursued to individual alleles with a set of allele-specific FNP markers, which helped to discover more specific alleles within the functional haplotypes. Despite this, there were three, two, and fourteen cultivars in the *Pid2*, *Pid3*, and *Pid4* categories, respectively, that were presumed to carry new types of alleles compared to the defined alleles (Table 1). This, in turn, indicated that each comprehensive FNP marker system was inclusive for finding unlimited new alleles, as it included the major FNPs at each locus. Collectively, the comprehensive FNP marker systems used in the present study were largely improved from those used in previous investigations, as almost of those were in the context of a working model (such as is the ‘specific marker(s) for specific alleles’ model) for allele mining. In contrast, the working model (the deeper allele mining model) used in the current study can be defined as a ‘comprehensive marker system for the whole locus/cluster’, which makes for an inclusive and comparable approach for discovering a series of new alleles, as in the three cases shown in the current study [30,31,33,36,44,45]. The outstanding merit of the comprehensive FNP marker system is advantageous for mining new alleles for breeding as well as for revealing the molecular mechanisms underlying the genetic divergence of the whole locus/cluster (Table 1).

### 4.2. The Pid Family Has Strictly Diverged into Xian and Geng Subspecies

Four alleles were detected at *Pid2*, of which the three functional ones were almost entirely restricted to *Xian* type cultivars while the null allele was only present in *Geng* type germplasm (Table 1). The distribution of alleles at *Pid3* was very similar: the four functional ones were harboured for the most part by *Xian* type entries and the null allele was common in the *Geng* genepool (Table 1). The latter result echoes a prior finding that the alleles of *Pid3* present in *Geng* type cultivars are pseudogenes [36,44]. In contrast, the distribution of alleles at *Pid4* featured five functional alleles which were shared evenly among the *Geng* type entries, with the null allele found only in *Xian* type ones (Table 1). It is notable that the well-known resistance alleles at the *Pi2*/*9*, *Pia*, and *Pita* loci/clusters have evenly diverged into both subspecies [37,38]. This may represent the first time that both *Xian* and *Geng* type resistance genes have been discovered and defined within individual cultivars through deeper allele mining with the comprehensive FNP marker system (Table S2). The data revealed by the FNP screen suggest a plausible genetic basis for the stable and broad blast resistance exhibited by the modern cultivars Digu, R207, Lu28S, Tianfeng B, R217, Zhonghua 11, Gumeizao 4, Moliruanzhan, Yuehesimiao, and Yuejingsimiao 2, in that all of these cultivars harbour a functional allele at each of the three *Pid* genes (Table S2). It might be truly expected that integration of both *Xian* and *Geng* resistance genes into upcoming cultivars would be one of the most promising ways to enlarge the genetic diversities of resistance genic resources, thereby withstanding ever-growing pressure from the pathogen across the *Xian* and *Geng* rice areas [2,5,10,11,13,36,44].

### 4.3. Rather Limited Genotypes of the Pid Family Are Effective in Both the Xian and Geng Rice Groups

By possessing three members of the *Pid* family, there would be seven total possible genotypes (combinations) (2^3^ − 1 = 7): *d2*, *d3*, *d4*; *d2*-*d3*, *d2*-*d4*, *d3*-*d4*, and *d2*-*d3*-*d4*, irrespective of specific alleles. However, only three genotypes, *d4*, *d2-d3*, and *d2-d3-d4*, were detected in the three panels consisting of 128 diversified rice germplasms (Table 1 and Table S2). The indication is therefore that rather limited genotypes of the *Pid* family have been integrated into both *Xian* and *Geng* rice cultivars in China. As all three members have strictly diverged into the two subspecies across the landrace and modern rice eras, *d2*-*d3* was centralized in the *Xian* group and *d4* in *Geng*, with both reaching overwhelming proportions, as was *d2*-*d3*-*d4* in the *Xian* group at a rather more moderate rate (Table 1). The genomic structure of the region harbouring the *Pid* family does not suggest any obvious barrier to local recombination (Figure S8); that is, there were four types of such barriers in the target genomic region: the key subspecies hybrid sterile gene cluster *S5* [46], the heading date gene *Hd1* [47], the photonasty gene *Se5* [48], and the centromere of rice chromosome 6 [49], all of which were far enough from the genic positions of the *Pid* family. In addition, genomic intervals among the three members were long enough to independently segregate each other in any particular genetic cross (Figure S8). Again, the *P2*/*9* cluster near the *Pid* family did not show any subspecific divergence in the same rice population tested in the current investigation (Figure S8) [37,38]. This in turn indicates that the subspecific divergence in the genomic region was specific to the *Pid* family.

One of the most likely genetic determinants leading to the establishment of such specific allelic and genotypic structures in the *Pid* family is due to the specific lineage(s) of the Chinese rice population; that is, the Chinese rice population has been derived from rather limited founder parents for its age. In reviewing the pedigrees of several Top-Ten cultivar types, including the general cultivars and F_1_ hybrid crosses, Liu [50] pointed out that in the specific lineage ‘Zhenzhuai 11-ZS97’ both were recognized as *Pid2*-ZS_*Pid3*-ZS, which has been central to Chinese *Xian* type rice breeding programs since the 1960s (see www.ricedata.cn/variety, accessed on 12 September 2020). The specific lineage perfectly addressed the questions of why there were two *Xian* type alleles with much higher rates in the respective allelic structures (*Pid2*-ZS with 71.4% (50/73) and *Pid3*-ZS with 86.3% (63/73)) and why there was not any single-gene genotype for *d2* or *d3* (with *d2*-*d3* being predominant among the three effective genotypes for a long time; see Table 1 and Table S2). That is, the unique allelic structures of the three members of the *Pid* family have been mainly constructed by the genotype, *d2*-*d3*, specifically *Pid2*-ZS_ *Pid3*-ZS, carried by the lineage in rice breeding programs in China after the 1960s. Thus, updating the lineage is again found to be the key to enlarging the genetic diversity of next-generation rice cultivars in China [2,5,36,44,50] (www.ricedata.cn/variety, accessed on 12 September 2020).

A further priority is to address the intriguing question of whether and why rather limited genotypes of the *Pid* family are predominant in other rice germplasm populations using both plant genetic resources and functional genomics approaches.

## 5. Conclusions

The study has demonstrated that all three members of the *Pid* family have been strictly diverged into *Xian* and *Geng* subspecies: *Pid2* and *Pid3* can be defined as *Xian* type resistance genes and *Pid4* as *Geng* type. Rather limited genotypes of the *Pid* family have been effective in both *Xian* and *Geng* rice groups, of which *Pid2*-ZS_*Pid3*-ZS is central to the Chinese rice population. This study has demonstrated the resistance functions of both *Pid2*-ZS and *Pid3*-ZS via their transgenic progenies.

## 6. Patents

Qinghua Pan, Xing Wang, Jinyan Wang, Li Wang, Ruipeng Chai, Ying Zhang, Ling Wang. A set of three critical and comprehensive FNP marker systems for deeper allele mining for rice blast resistance genes *Pid2*, *Pid3*, and *Pid4* (20211102878.7, pending on 3 September 2021).

## Figures and Tables

**Table 1:** Distribution of alleles and genotypes of the *Pid* family in the *Xian* and *Geng* rice gene pools.

Alleles vs. Genotypes	*Xian* Group (*a*, Presence/*b*, Absence)	*Geng* Group (*c*, Presence/*d*, Absence)	*χ*^2^ for Homogeneity ^a^
Alleles			
*Pid2* alleles	69/1	4/54	105.07 ***
*Pid2*-ZS	49	1	
*Pid2*-DIG	17	3	
*Pid2*-new	3	0	
*Pid2*-null	1	54	
*Pid3* alleles	69/1	4/54	105.07 ***
*Pid3*-ZS	60	3	
*Pid3*-DIG	5	1	
*Pid3*-TTP	2	0	
*Pid3*-new	2	0	
*Pid3*-null	1	54	
*Pid4* alleles	19/51	56/2	60.16 ***
*Pid4*-NPB	1	21	
*Pid4*-SN	3	16	
*Pid4*-CO	4	14	
*Pid4*-new	9	5	
*Pid4*-DIG	2	0	
*Pid4*-null	51	2	
Genotypes			
*d4*	1/69	54/4	105.07 ***
*d2-d3*	51/19	2/56	60.16 ***
*d2-d3-d4*	18/52	2/56	10.30 **

^a^*χ*^2^ homogeneity test to determine whether the paired *Pid* alleles varied between the two genepools. Calculations based on the formula,$\;\chi^{2}=\frac{N{\left[\left|\mathrm{ad}-\mathrm{bc}\right|-\left(\frac{1}{2}\right)N\right]}^{2}}{\left(a+b)(c+d)(a+c)(b+d\right)}$, where ** and *** represent the paired *Pid* alleles and genotypes differing significantly between the *Xian* and *Geng* genepools (*p* < 0.01 and *p* < 0.001, respectively; df = 1). The frequency of the presence and absence of each allele/genotype in the *Xian* genepool is provided by *a* and *b*, respectively, and in the *Geng* genepool by *c* and *d*, respectively. *N* denotes the total number of alleles/genotypes detected for each *Pid* gene/genotype.

## Data Availability

Not applicable.

## Supplementary Materials

The following supporting information can be downloaded at: https://www.mdpi.com/article/10.3390/genes13050891/s1, Figure S1: The *Pid2* CDSs harboured by the 15 reference rice cultivars; Figure S2: Allele mining of the *Pid2* alleles in the additional *Xian* and *Geng* rice cultivar panels; Figure S3: The *Pid3* CDSs harboured by the 15 reference rice cultivars; Figure S4: Allele mining of the *Pid3* alleles in the additional *Xian* and *Geng* rice cultivar panels; Figure S5: The *Pid4* genomic sequences harboured by the 13 reference rice cultivars; Figure S6: Allele mining of the *Pid4* alleles in the additional *Xian* and *Geng* rice cultivar panels; Figure S7: Validation of resistance function of two paired alleles of the *Pid* family; Figure S8: The genomic context of the three key genotypes of the *Pid* family detected in the present study; Table S1: The three comprehensive FNP marker systems for deeper allele mining, and the primers and markers for transformation test of the *Pid* family; Table S2: Two sets of rice cultivars for control and three panels of rice cultivars for allele mining used in the present study.
