# Supplementary material for: The Pid Family Has Been Diverged into Xian and Geng Type Resistance Genes against Rice Blast Disease
Source: Genes (Basel). 2022 May 17;13(5):891. doi: 10.3390/genes13050891 (PMC9141787; doi:10.3390/genes13050891)
Supplement: Supplementary file 1 [file genes-13-00891-s001.zip › genes-1711621-supplementary/Figure S2. Pid2 identities in GD-HLJ.pptx]

## Slide 1
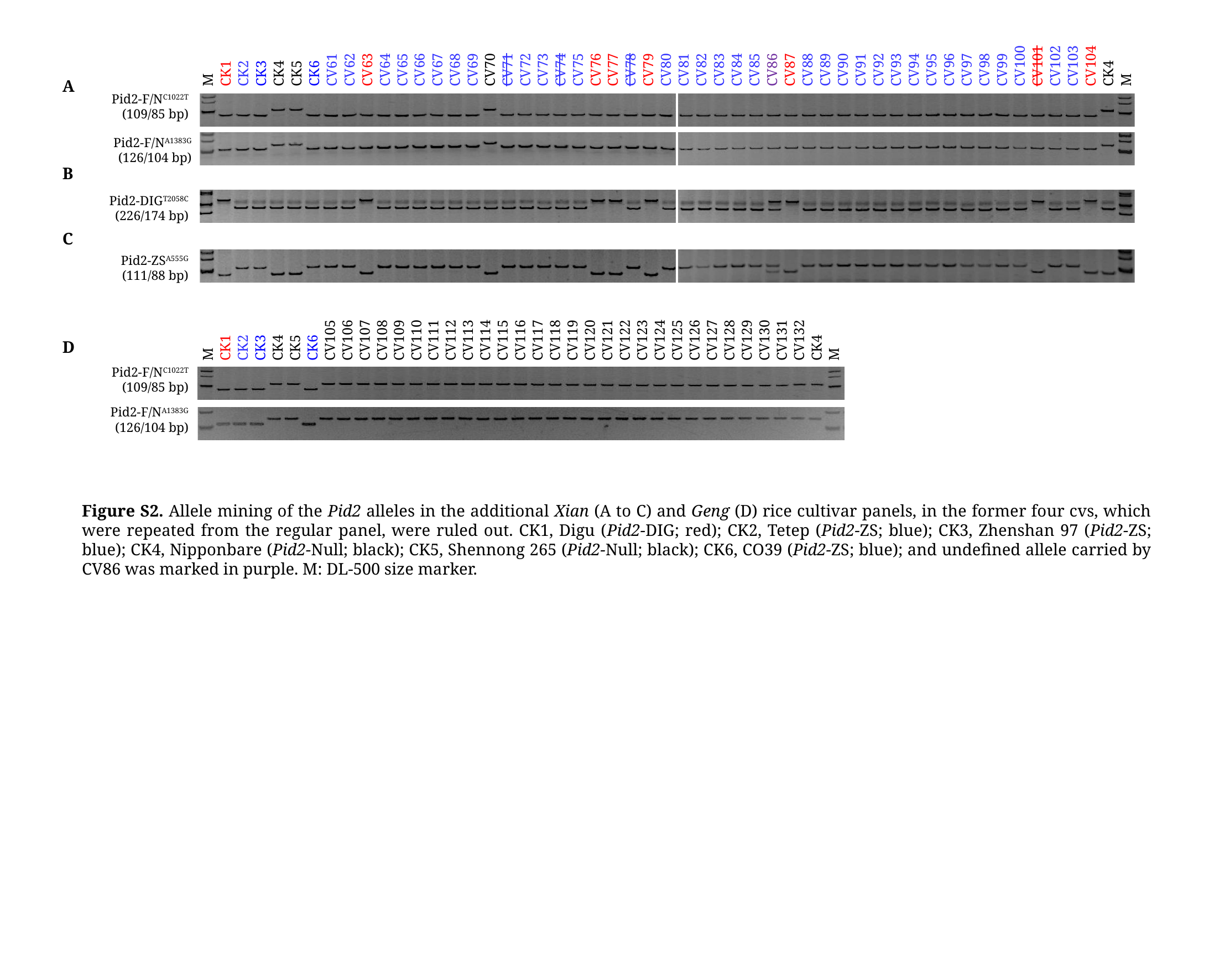

| M | CK1 | CK2 | CK3 | CK4 | CK5 | CK6 | CV61 | CV62 | CV63 | CV64 | CV65 | CV66 | CV67 | CV68 | CV69 | CV70 | CV71 | CV72 | CV73 | CV74 | CV75 | CV76 | CV77 | CV78 | CV79 | CV80 | CV81 | CV82 | CV83 | CV84 | CV85 | CV86 | CV87 | CV88 | CV89 | CV90 | CV91 | CV92 | CV93 | CV94 | CV95 | CV96 | CV97 | CV98 | CV99 | CV100 | CV101 | CV102 | CV103 | CV104 | CK4 | M |
| --- | --- | --- | --- | --- | --- | --- | --- | --- | --- | --- | --- | --- | --- | --- | --- | --- | --- | --- | --- | --- | --- | --- | --- | --- | --- | --- | --- | --- | --- | --- | --- | --- | --- | --- | --- | --- | --- | --- | --- | --- | --- | --- | --- | --- | --- | --- | --- | --- | --- | --- | --- | --- |
A
Pid2-F/NC1022T
(109/85 bp)
Pid2-F/NA1383G
(126/104 bp)
B
Pid2-DIGT2058C
(226/174 bp)
C
Pid2-ZSA555G
(111/88 bp)
| M | CK1 | CK2 | CK3 | CK4 | CK5 | CK6 | CV105 | CV106 | CV107 | CV108 | CV109 | CV110 | CV111 | CV112 | CV113 | CV114 | CV115 | CV116 | CV117 | CV118 | CV119 | CV120 | CV121 | CV122 | CV123 | CV124 | CV125 | CV126 | CV127 | CV128 | CV129 | CV130 | CV131 | CV132 | CK4 | M |
| --- | --- | --- | --- | --- | --- | --- | --- | --- | --- | --- | --- | --- | --- | --- | --- | --- | --- | --- | --- | --- | --- | --- | --- | --- | --- | --- | --- | --- | --- | --- | --- | --- | --- | --- | --- | --- |
D
Pid2-F/NC1022T
(109/85 bp)
Pid2-F/NA1383G
(126/104 bp)
Figure S2. Allele mining of the Pid2 alleles in the additional Xian (A to C) and Geng (D) rice cultivar panels, in the former four cvs, which were repeated from the regular panel, were ruled out. CK1, Digu (Pid2-DIG; red); CK2, Tetep (Pid2-ZS; blue); CK3, Zhenshan 97 (Pid2-ZS; blue); CK4, Nipponbare (Pid2-Null; black); CK5, Shennong 265 (Pid2-Null; black); CK6, CO39 (Pid2-ZS; blue); and undefined allele carried by CV86 was marked in purple. M: DL-500 size marker.
