# Supplementary material for: The Pid Family Has Been Diverged into Xian and Geng Type Resistance Genes against Rice Blast Disease
Source: Genes (Basel). 2022 May 17;13(5):891. doi: 10.3390/genes13050891 (PMC9141787; doi:10.3390/genes13050891)
Supplement: Supplementary file 1 [file genes-13-00891-s001.zip › genes-1711621-supplementary/Figure S3. Pid3 CDS.pptx]

## Slide 1
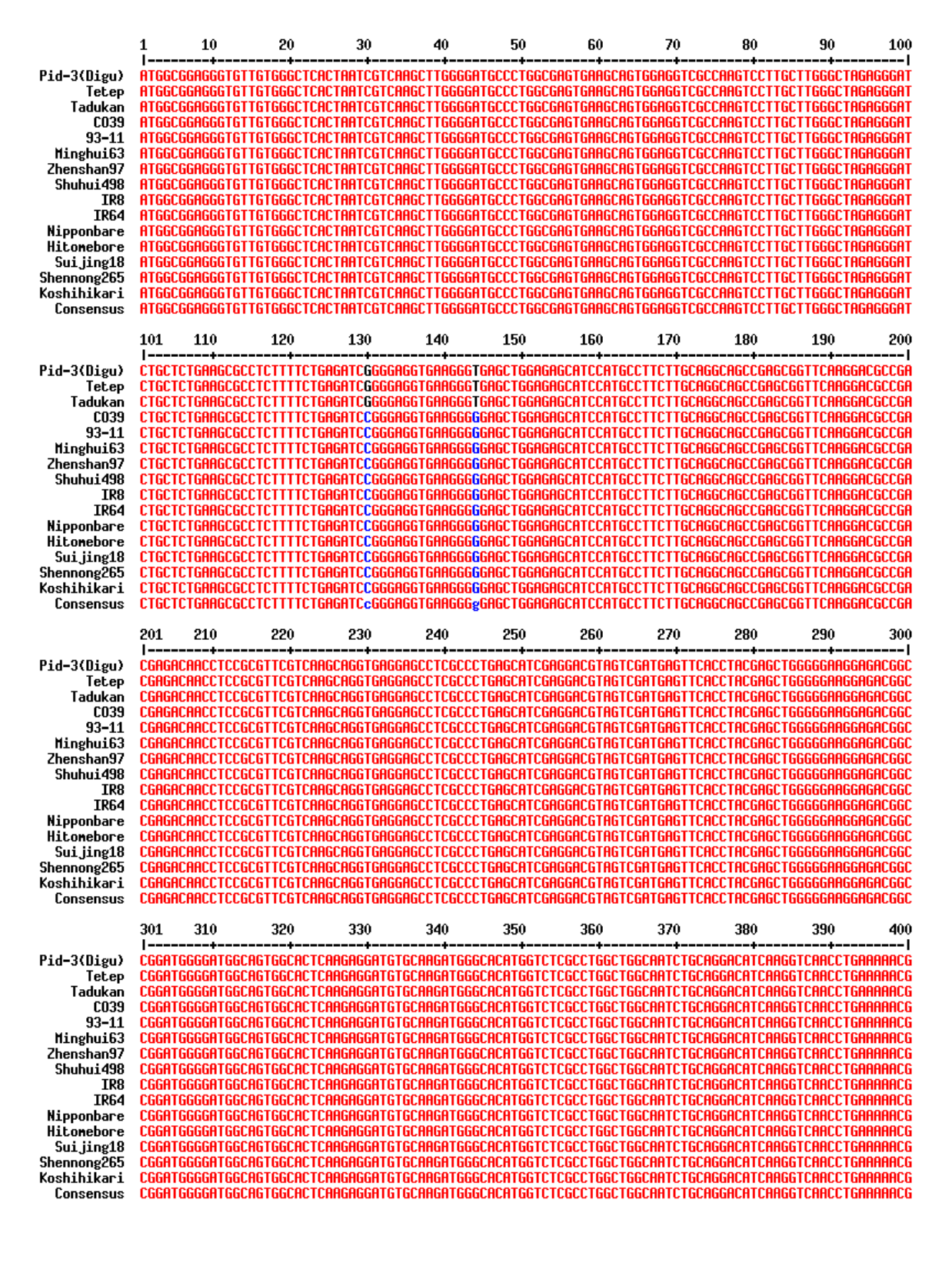

## Slide 2
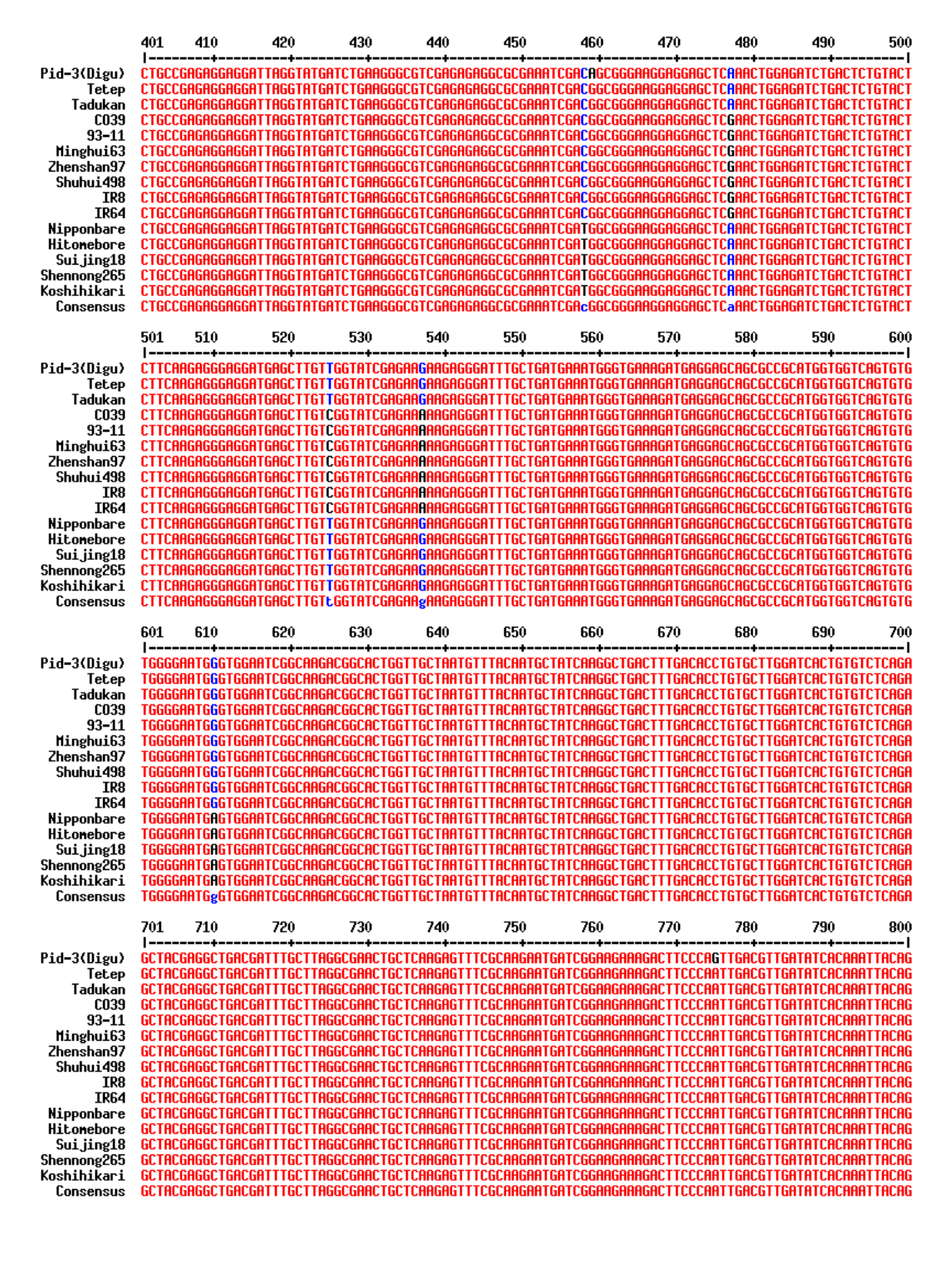

## Slide 3
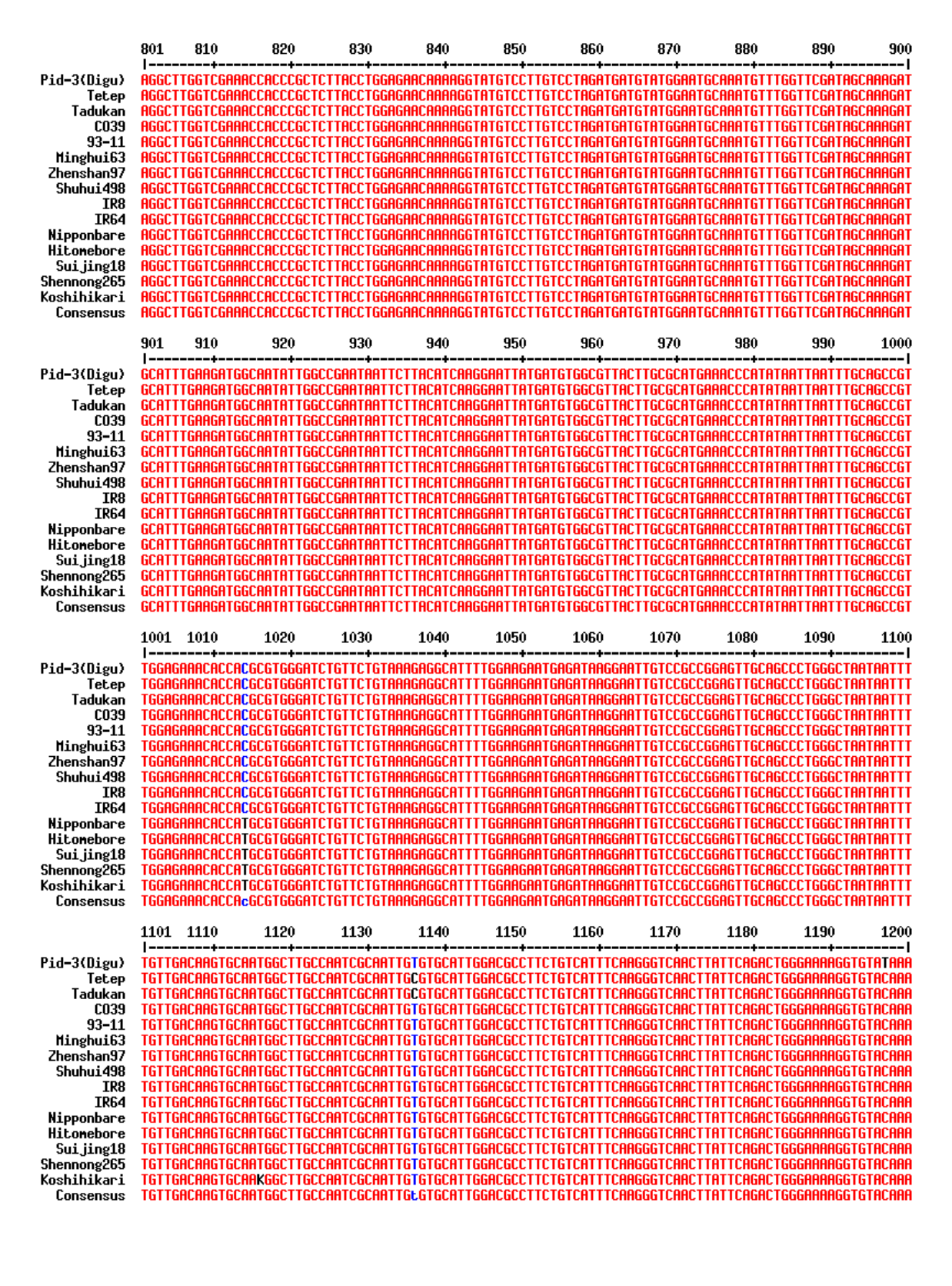

## Slide 4
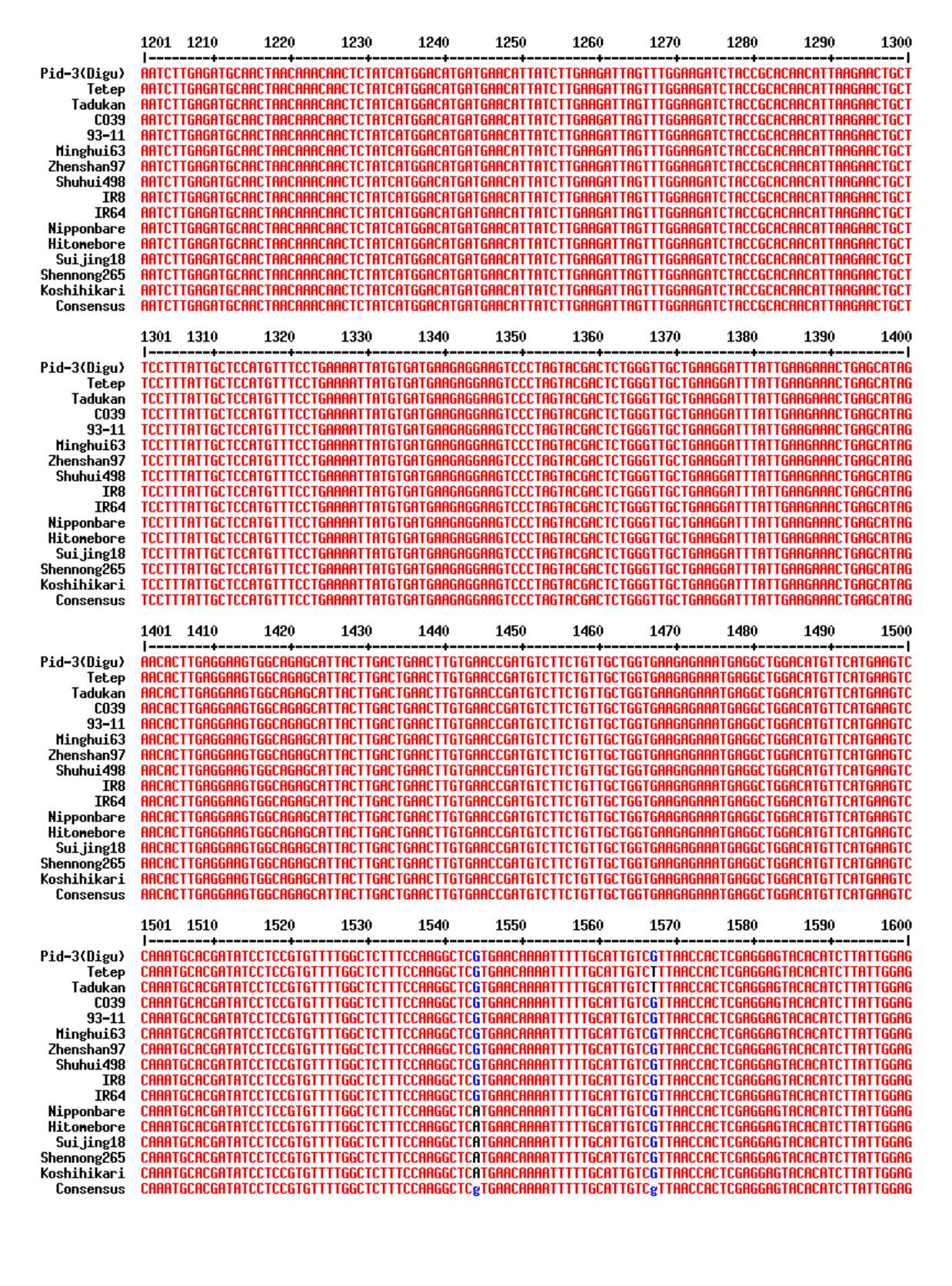

## Slide 5
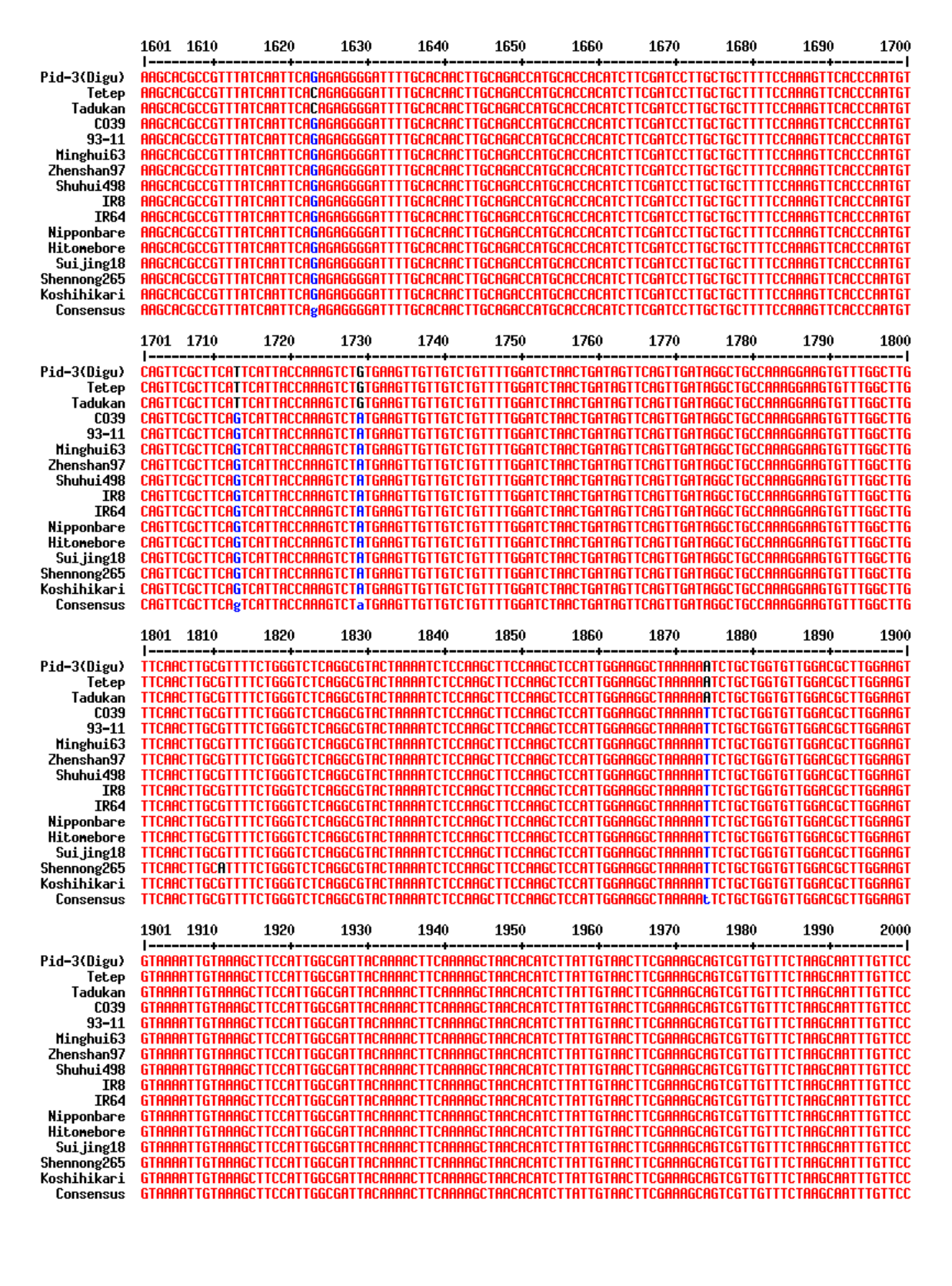

## Slide 6
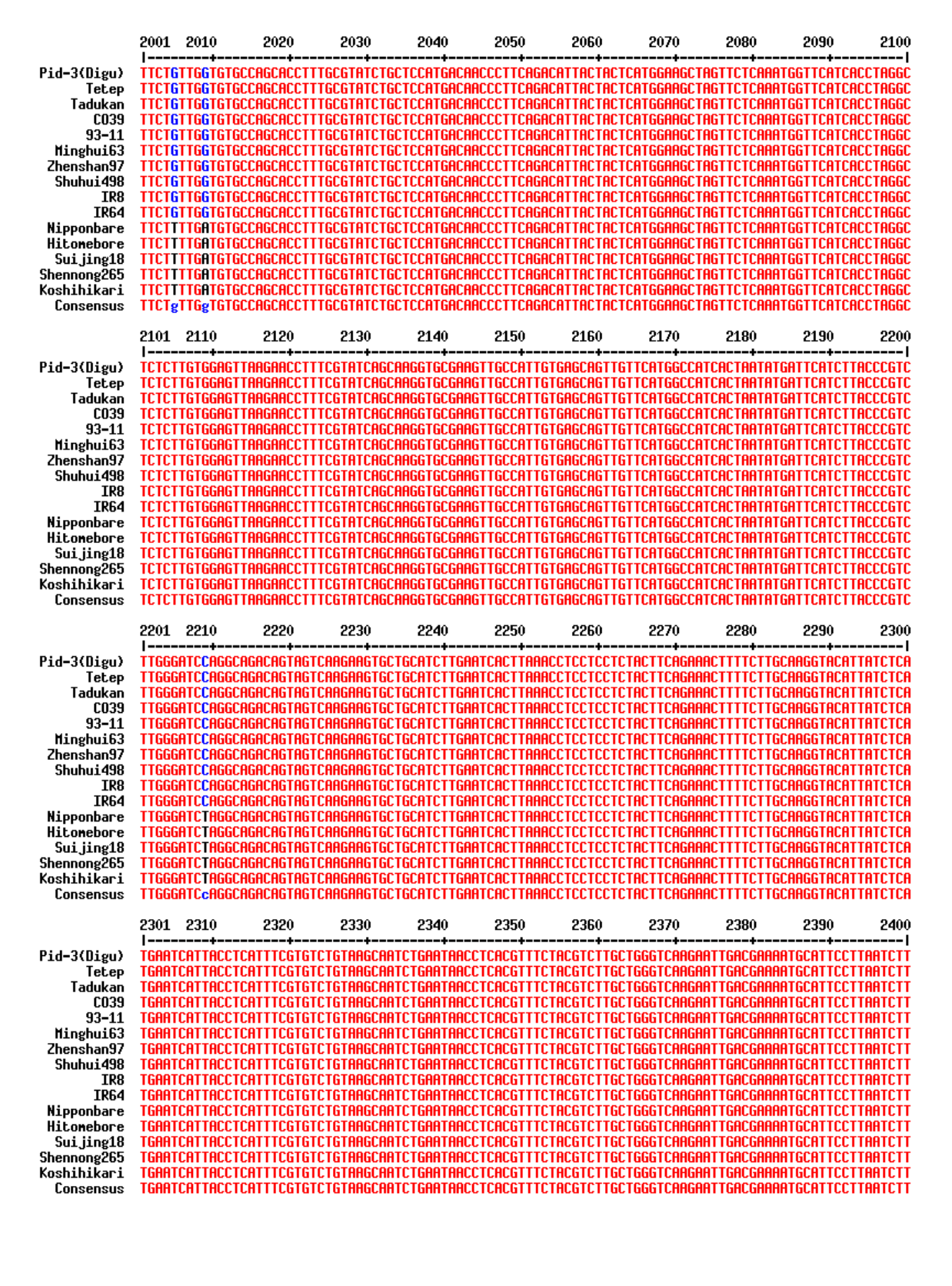

## Slide 7
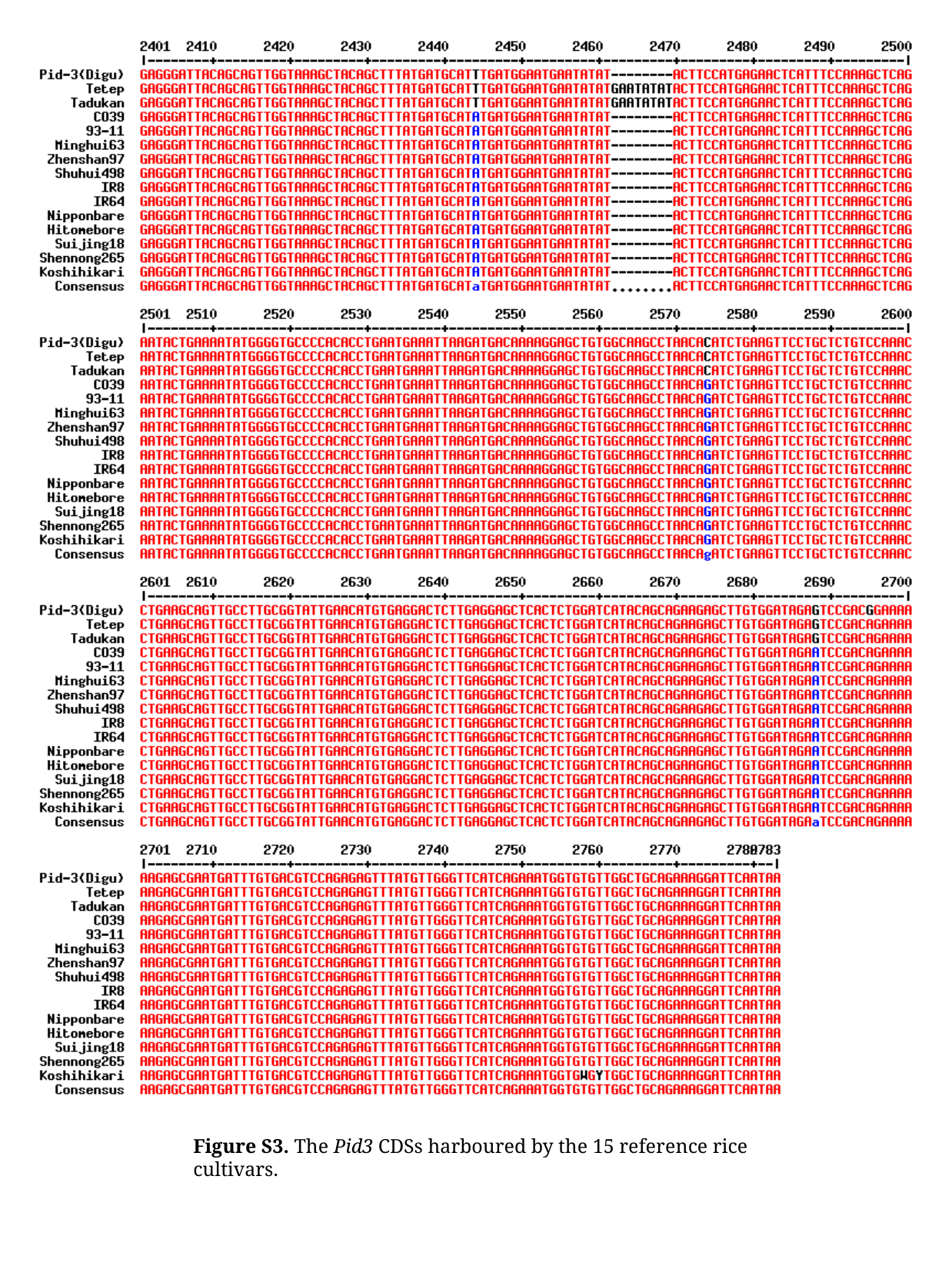

Figure S3. The Pid3 CDSs harboured by the 15 reference rice cultivars.
