# Supplementary material for: The Pid Family Has Been Diverged into Xian and Geng Type Resistance Genes against Rice Blast Disease
Source: Genes (Basel). 2022 May 17;13(5):891. doi: 10.3390/genes13050891 (PMC9141787; doi:10.3390/genes13050891)
Supplement: Supplementary file 1 [file genes-13-00891-s001.zip › genes-1711621-supplementary/Figure S4. Pid3 identities in GD-HLJ.pptx]

## Slide 1
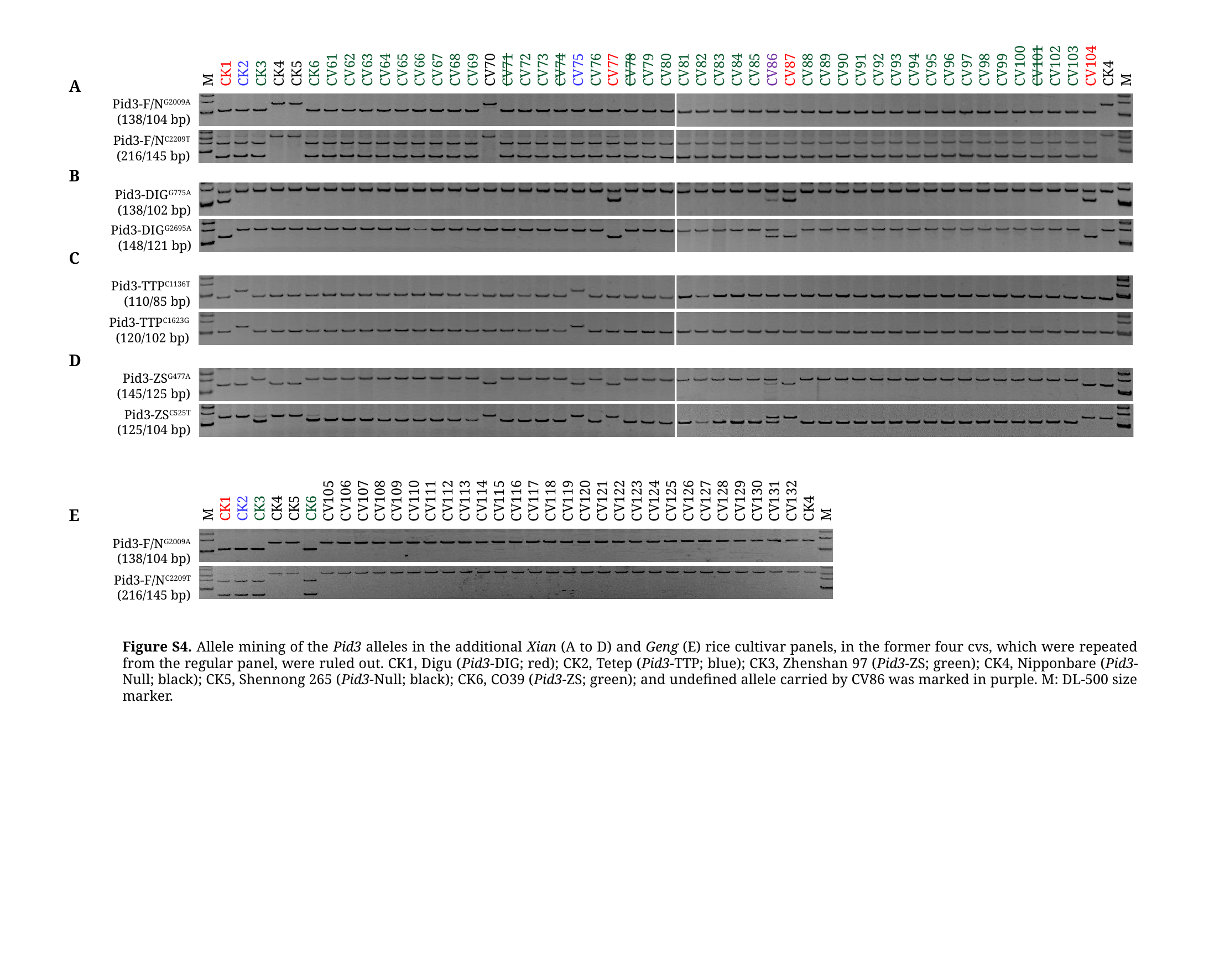

| M | CK1 | CK2 | CK3 | CK4 | CK5 | CK6 | CV61 | CV62 | CV63 | CV64 | CV65 | CV66 | CV67 | CV68 | CV69 | CV70 | CV71 | CV72 | CV73 | CV74 | CV75 | CV76 | CV77 | CV78 | CV79 | CV80 | CV81 | CV82 | CV83 | CV84 | CV85 | CV86 | CV87 | CV88 | CV89 | CV90 | CV91 | CV92 | CV93 | CV94 | CV95 | CV96 | CV97 | CV98 | CV99 | CV100 | CV101 | CV102 | CV103 | CV104 | CK4 | M |
| --- | --- | --- | --- | --- | --- | --- | --- | --- | --- | --- | --- | --- | --- | --- | --- | --- | --- | --- | --- | --- | --- | --- | --- | --- | --- | --- | --- | --- | --- | --- | --- | --- | --- | --- | --- | --- | --- | --- | --- | --- | --- | --- | --- | --- | --- | --- | --- | --- | --- | --- | --- | --- |
A
Pid3-F/NG2009A
(138/104 bp)
Pid3-F/NC2209T
(216/145 bp)
B
Pid3-DIGG775A
(138/102 bp)
Pid3-DIGG2695A
(148/121 bp)
C
Pid3-TTPC1136T
(110/85 bp)
Pid3-TTPC1623G
(120/102 bp)
D
Pid3-ZSG477A
(145/125 bp)
Pid3-ZSC525T
(125/104 bp)
| M | CK1 | CK2 | CK3 | CK4 | CK5 | CK6 | CV105 | CV106 | CV107 | CV108 | CV109 | CV110 | CV111 | CV112 | CV113 | CV114 | CV115 | CV116 | CV117 | CV118 | CV119 | CV120 | CV121 | CV122 | CV123 | CV124 | CV125 | CV126 | CV127 | CV128 | CV129 | CV130 | CV131 | CV132 | CK4 | M |
| --- | --- | --- | --- | --- | --- | --- | --- | --- | --- | --- | --- | --- | --- | --- | --- | --- | --- | --- | --- | --- | --- | --- | --- | --- | --- | --- | --- | --- | --- | --- | --- | --- | --- | --- | --- | --- |
E
Pid3-F/NG2009A
(138/104 bp)
Pid3-F/NC2209T
(216/145 bp)
Figure S4. Allele mining of the Pid3 alleles in the additional Xian (A to D) and Geng (E) rice cultivar panels, in the former four cvs, which were repeated from the regular panel, were ruled out. CK1, Digu (Pid3-DIG; red); CK2, Tetep (Pid3-TTP; blue); CK3, Zhenshan 97 (Pid3-ZS; green); CK4, Nipponbare (Pid3-Null; black); CK5, Shennong 265 (Pid3-Null; black); CK6, CO39 (Pid3-ZS; green); and undefined allele carried by CV86 was marked in purple. M: DL-500 size marker.
