# Supplementary material for: The Pid Family Has Been Diverged into Xian and Geng Type Resistance Genes against Rice Blast Disease
Source: Genes (Basel). 2022 May 17;13(5):891. doi: 10.3390/genes13050891 (PMC9141787; doi:10.3390/genes13050891)
Supplement: Supplementary file 1 [file genes-13-00891-s001.zip › genes-1711621-supplementary/Figure S6. Pid4 identities in GD-HLJ.pptx]

## Slide 1
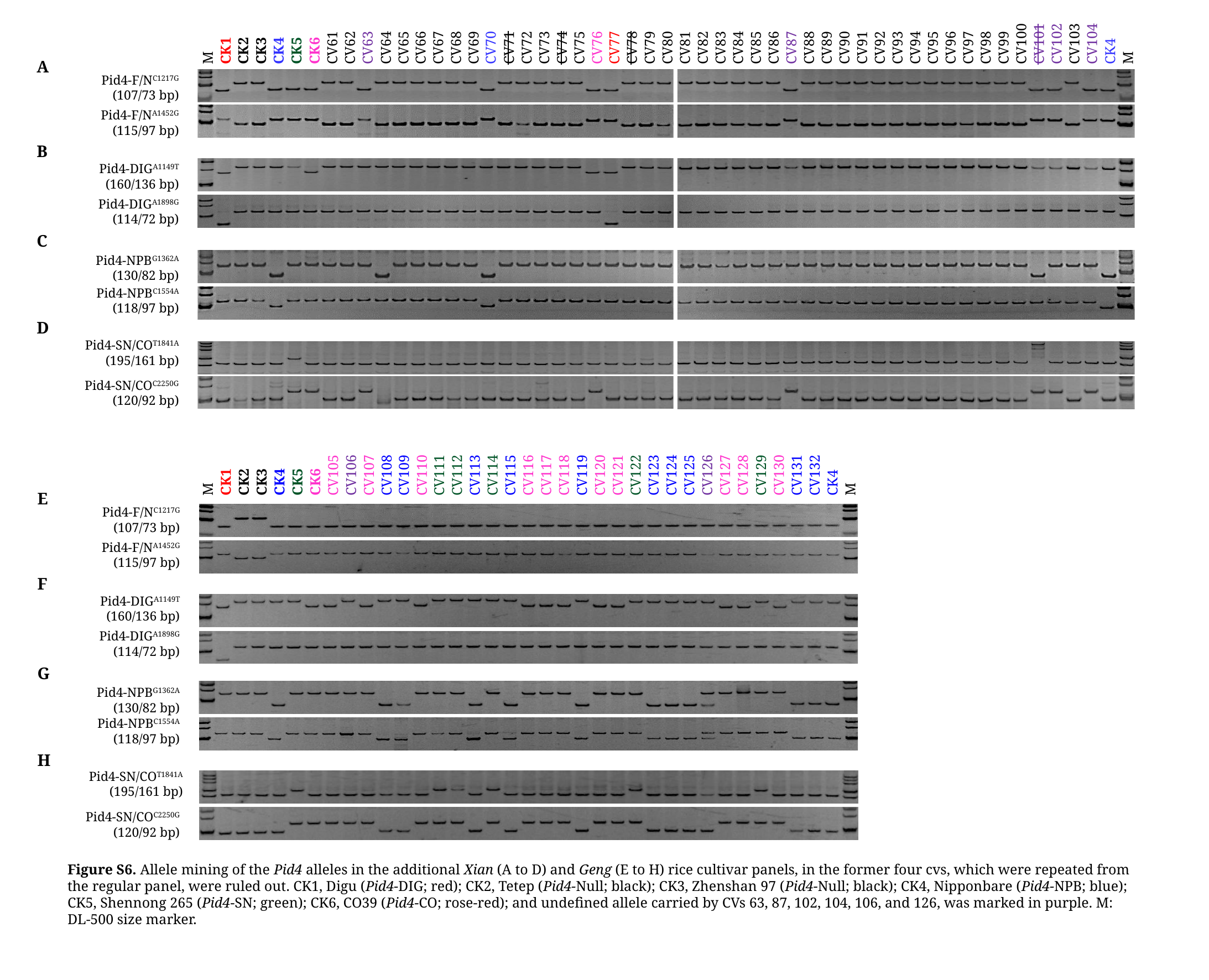

| M | CK1 | CK2 | CK3 | CK4 | CK5 | CK6 | CV61 | CV62 | CV63 | CV64 | CV65 | CV66 | CV67 | CV68 | CV69 | CV70 | CV71 | CV72 | CV73 | CV74 | CV75 | CV76 | CV77 | CV78 | CV79 | CV80 | CV81 | CV82 | CV83 | CV84 | CV85 | CV86 | CV87 | CV88 | CV89 | CV90 | CV91 | CV92 | CV93 | CV94 | CV95 | CV96 | CV97 | CV98 | CV99 | CV100 | CV101 | CV102 | CV103 | CV104 | CK4 | M |
| --- | --- | --- | --- | --- | --- | --- | --- | --- | --- | --- | --- | --- | --- | --- | --- | --- | --- | --- | --- | --- | --- | --- | --- | --- | --- | --- | --- | --- | --- | --- | --- | --- | --- | --- | --- | --- | --- | --- | --- | --- | --- | --- | --- | --- | --- | --- | --- | --- | --- | --- | --- | --- |
A
Pid4-F/NC1217G
(107/73 bp)
Pid4-F/NA1452G
(115/97 bp)
B
Pid4-DIGA1149T
(160/136 bp)
Pid4-DIGA1898G
(114/72 bp)
C
Pid4-NPBG1362A
(130/82 bp)
Pid4-NPBC1554A
(118/97 bp)
D
Pid4-SN/COT1841A
(195/161 bp)
Pid4-SN/COC2250G
(120/92 bp)
| M | CK1 | CK2 | CK3 | CK4 | CK5 | CK6 | CV105 | CV106 | CV107 | CV108 | CV109 | CV110 | CV111 | CV112 | CV113 | CV114 | CV115 | CV116 | CV117 | CV118 | CV119 | CV120 | CV121 | CV122 | CV123 | CV124 | CV125 | CV126 | CV127 | CV128 | CV129 | CV130 | CV131 | CV132 | CK4 | M |
| --- | --- | --- | --- | --- | --- | --- | --- | --- | --- | --- | --- | --- | --- | --- | --- | --- | --- | --- | --- | --- | --- | --- | --- | --- | --- | --- | --- | --- | --- | --- | --- | --- | --- | --- | --- | --- |
E
Pid4-F/NC1217G
(107/73 bp)
Pid4-F/NA1452G
(115/97 bp)
F
Pid4-DIGA1149T
(160/136 bp)
Pid4-DIGA1898G
(114/72 bp)
G
Pid4-NPBG1362A
(130/82 bp)
Pid4-NPBC1554A
(118/97 bp)
H
Pid4-SN/COT1841A
(195/161 bp)
Pid4-SN/COC2250G
(120/92 bp)
Figure S6. Allele mining of the Pid4 alleles in the additional Xian (A to D) and Geng (E to H) rice cultivar panels, in the former four cvs, which were repeated from the regular panel, were ruled out. CK1, Digu (Pid4-DIG; red); CK2, Tetep (Pid4-Null; black); CK3, Zhenshan 97 (Pid4-Null; black); CK4, Nipponbare (Pid4-NPB; blue); CK5, Shennong 265 (Pid4-SN; green); CK6, CO39 (Pid4-CO; rose-red); and undefined allele carried by CVs 63, 87, 102, 104, 106, and 126, was marked in purple. M: DL-500 size marker.
