# Supplementary material for: The Pid Family Has Been Diverged into Xian and Geng Type Resistance Genes against Rice Blast Disease
Source: Genes (Basel). 2022 May 17;13(5):891. doi: 10.3390/genes13050891 (PMC9141787; doi:10.3390/genes13050891)
Supplement: Supplementary file 1 [file genes-13-00891-s001.zip › genes-1711621-supplementary/Figure S7. Pid transgenic plants.pptx]

## Slide 1
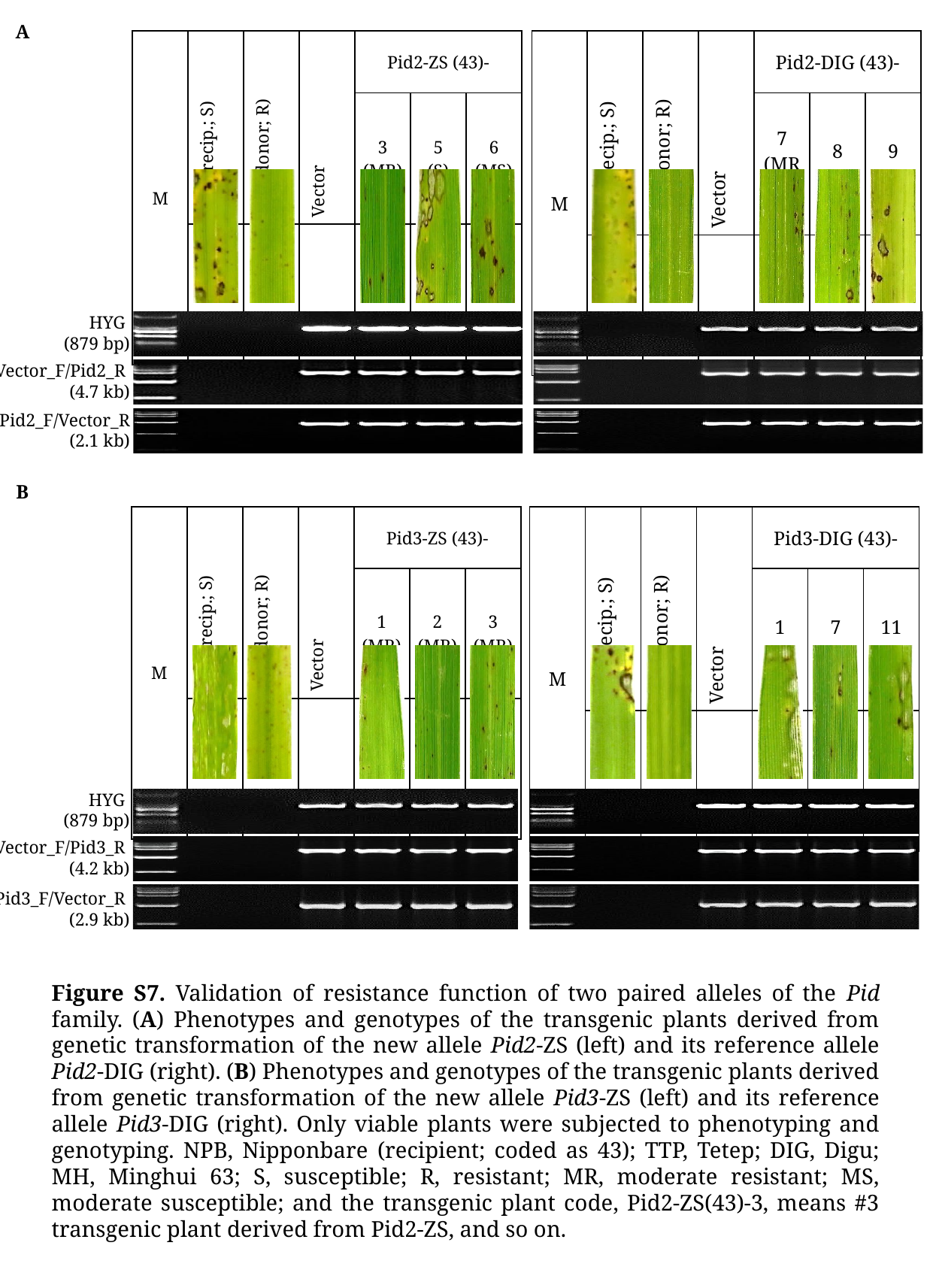

A
| M | NPB (recip.; S) | TTP (donor; R) | Vector | Pid2-ZS (43)- | | |
| --- | --- | --- | --- | --- | --- | --- |
| | | | | 3 (MR) | 5 (S) | 6 (MS) |
| | | | | | | |
| M | NPB (recip.; S) | DIG (donor; R) | Vector | Pid2-DIG (43)- | | |
| --- | --- | --- | --- | --- | --- | --- |
| | | | | 7 (MR) | 8 (MS) | 9 (MS) |
| | | | | | | |
HYG
(879 bp)
Vector_F/Pid2_R
(4.7 kb)
Pid2_F/Vector_R (2.1 kb)
B
| M | NPB (recip.; S) | MH (donor; R) | Vector | Pid3-ZS (43)- | | |
| --- | --- | --- | --- | --- | --- | --- |
| | | | | 1 (MR) | 2 (MR) | 3 (MR) |
| | | | | | | |
| M | NPB (recip.; S) | DIG (donor; R) | Vector | Pid3-DIG (43)- | | |
| --- | --- | --- | --- | --- | --- | --- |
| | | | | 1 (S) | 7 (MS) | 11 (S) |
| | | | | | | |
HYG
(879 bp)
Vector_F/Pid3_R
(4.2 kb)
Pid3_F/Vector_R
(2.9 kb)
Figure S7. Validation of resistance function of two paired alleles of the Pid family. (A) Phenotypes and genotypes of the transgenic plants derived from genetic transformation of the new allele Pid2-ZS (left) and its reference allele Pid2-DIG (right). (B) Phenotypes and genotypes of the transgenic plants derived from genetic transformation of the new allele Pid3-ZS (left) and its reference allele Pid3-DIG (right). Only viable plants were subjected to phenotyping and genotyping. NPB, Nipponbare (recipient; coded as 43); TTP, Tetep; DIG, Digu; MH, Minghui 63; S, susceptible; R, resistant; MR, moderate resistant; MS, moderate susceptible; and the transgenic plant code, Pid2-ZS(43)-3, means #3 transgenic plant derived from Pid2-ZS, and so on.
