# Supplementary material for: The Pid Family Has Been Diverged into Xian and Geng Type Resistance Genes against Rice Blast Disease
Source: Genes (Basel). 2022 May 17;13(5):891. doi: 10.3390/genes13050891 (PMC9141787; doi:10.3390/genes13050891)
Supplement: Supplementary file 1 [file genes-13-00891-s001.zip › genes-1711621-supplementary/Figure S8. Haplotype structures.pptx]

## Slide 1
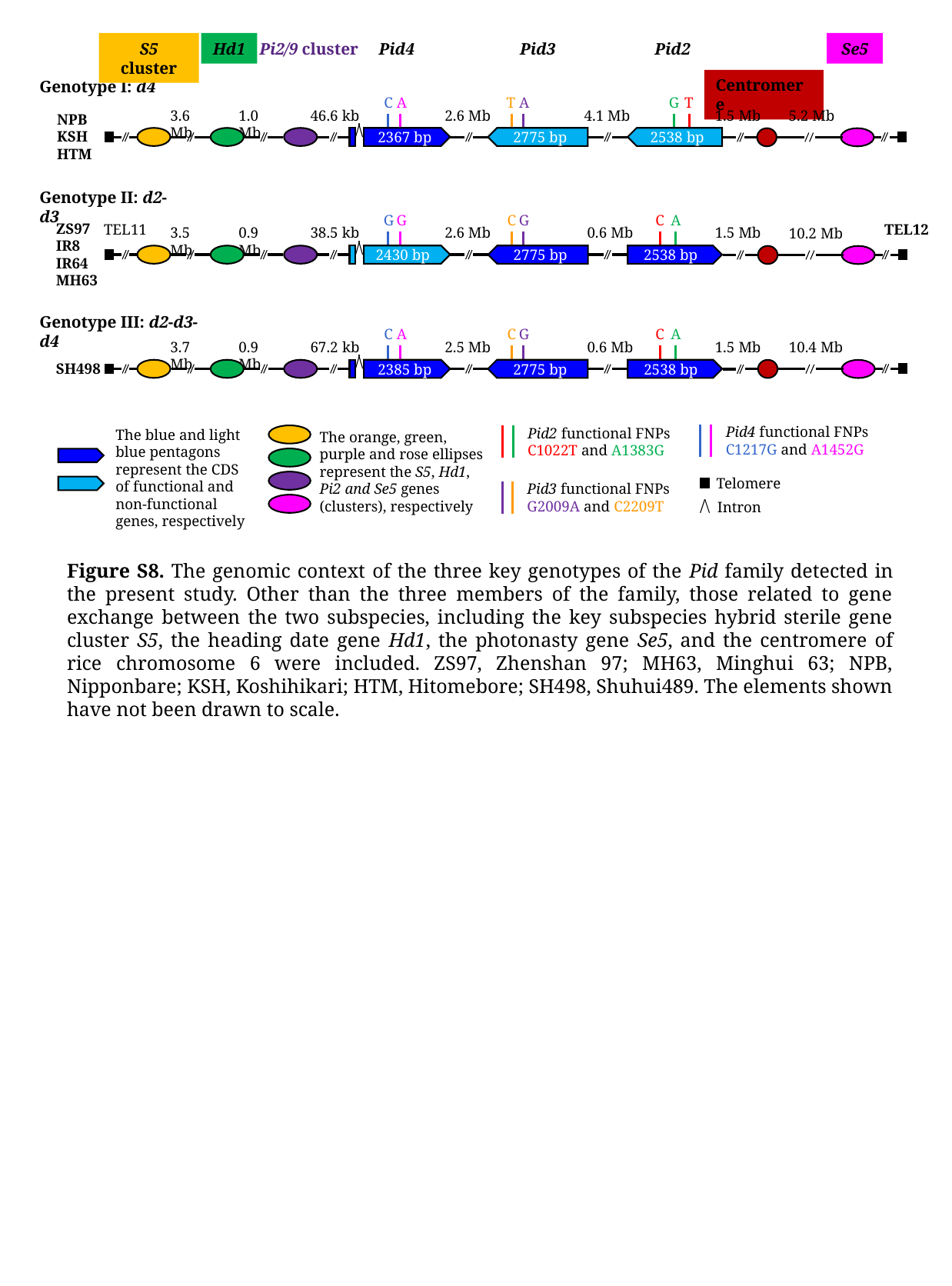

Hd1
S5 cluster
Pi2/9 cluster
Pid4
Pid3
Pid2
Se5
Centromere
Genotype I: d4
C
A
T
A
G
T
3.6 Mb
1.0 Mb
46.6 kb
2.6 Mb
4.1 Mb
1.5 Mb
5.2 Mb
NPB
KSH
HTM
2367 bp
2775 bp
2538 bp
Genotype II: d2-d3
G
G
C
G
C
A
ZS97
IR8
IR64
MH63
TEL11
TEL12
3.5 Mb
0.9 Mb
38.5 kb
2.6 Mb
0.6 Mb
1.5 Mb
10.2 Mb
2430 bp
2775 bp
2538 bp
Genotype III: d2-d3-d4
C
A
C
G
C
A
10.4 Mb
3.7 Mb
0.9 Mb
67.2 kb
2.5 Mb
0.6 Mb
1.5 Mb
SH498
2385 bp
2775 bp
2538 bp
Pid4 functional FNPs C1217G and A1452G
Pid2 functional FNPs C1022T and A1383G
The blue and light blue pentagons represent the CDS of functional and non-functional genes, respectively
The orange, green, purple and rose ellipses represent the S5, Hd1, Pi2 and Se5 genes (clusters), respectively
Telomere
Pid3 functional FNPs G2009A and C2209T
Intron
Figure S8. The genomic context of the three key genotypes of the Pid family detected in the present study. Other than the three members of the family, those related to gene exchange between the two subspecies, including the key subspecies hybrid sterile gene cluster S5, the heading date gene Hd1, the photonasty gene Se5, and the centromere of rice chromosome 6 were included. ZS97, Zhenshan 97; MH63, Minghui 63; NPB, Nipponbare; KSH, Koshihikari; HTM, Hitomebore; SH498, Shuhui489. The elements shown have not been drawn to scale.
