# Supplementary material for: The Pid Family Has Been Diverged into Xian and Geng Type Resistance Genes against Rice Blast Disease
Source: Genes (Basel). 2022 May 17;13(5):891. doi: 10.3390/genes13050891 (PMC9141787; doi:10.3390/genes13050891)
Supplement: Supplementary file 1 [file genes-13-00891-s001.zip › genes-1711621-supplementary/Table S1. Primers & Markers.pdf]

Table S1. The three comprehensive FNP marker systems for deeper allele mining, and the primers and markers for transformation test of the *Pid* family.

Gene (accession number) / Marker name / Primer sequence (5′→3′) [a]	Annealing temp (°C) [b]	Restriction enzyme (°C) [c]	Expected size (bp) [d]
**Set I. *Pid2* (FJ915121.1)**			
**Pid2-F/N^{C1022T}**	62	*Taq* I (65)	109/85
F: acagttccggcagactcttgtgacat			
R: agtttgcaaacgagccgagggtc			
**Pid2-F/N^{A1383G}**	56	*Mlu* I (37)	126/104
F: ggaatacaactcgtttcgcatct			
R: ctatgatagccaaagttacgcg			
**Pid2-DIG^{T2058C}**	58	*Hha* I (37)	226/174
F: agttgatgaccagggagcaga			
R: ccttcctccagcttcttgaa			
**Pid2-ZS^{A555G}**	53	*Hpa* II (37)	111/88
F: gccacctctatgcaactactg			
R: accagacagaagagtgtctgc			
**Set II. *Pid3* (FJ745364.1)**			
**Pid3-F/N^{G2009A}**	58	*Bsl* I (55)	138/104
F: gcagtcgttgtttctaagcaatttg			
R: cacaagagagcctaggtgatgaac			
**Pid3-F/N^{C2209T}**	62	*Bam* HI (37)	216/145
F: aaggtgcgaagttgccattgt			
R: cgtgaggttattcagattgcttacag			
**Pid3-DIG^{G775A}**	56	*Bsr* I (65)	138/102
F: aagagtttcgcaagaatgatcgg			
R: cattccatacatcatctaggacaagg			
**Pid3-DIG^{G2695A}**	58	*Hpy* 99I (37)	148/121
F: atctgaagttcctgctctgtccaa			
R: acgtcacaaatcattcgctct			
**Pid3-TTP^{C1136T}**	57	*Apa* LI (37)	110/85
F: aatgagataaggaattgtccgccg			
R: aatgacagaaggcgtccaatgtgc			
**Pid3-TTP^{C1623G}**	60	*Dde* I (37)	120/102
F: agcacgccgtttatcaactca			
R: ggtaatgactgaagcgaactg			
**Pid3-ZS^{G477A}**	60	*Mse* I (37)	145/125
dF: gayrgcgggaaggaggagctt			
R: cacactgaccaccatgcggc			
**Pid3-ZS^{C525T}**	62	*Hpa* II (37)	125/104

F: caagagggaggatgagcttgc			
R: ccgtcttgccgattccac			
**Set III. *Pid4* (MG839283.1)**			
**Pid4-F/N^{C1217G}**	57	*Mnl* I (37)	107/73
F: cataattcaaaactgtgcttatccct			
R: cctcataacttttatttgattttctca			
**Pid4-F/N^{A1452G}**	60	*Bsp* HI (37)	115/97
F: gcagctatcttcagggctcat			
R: gctaaggtacagaaaacatggct			
**Pid4-DIG^{A1149T}**	53	*Xba* I (37)	160/136
F: ttaatgaatctgtttttcctgactctag			
R: gattttctcagcagcaaatgtttagc			
**Pid4-DIG^{A1898G}**	62	*Bsr* I (65)	114/72
dF: gggagacaggaktcawacctaagc			
R1: gagcatccttagctgtggtgaac			
R2: gatcatccttagttgggacggac			
**Pid4-NPB^{G1362A}**	59	*Aci* I (37)	130/82
F: aggctgagaaggtgtttgata			
R: gaagatagctgcagagacagt			
**Pid4-NPB^{C1554A}**	53	*Aci* I (37)	118/97
F1: tttctgtaccttagcattttccg			
F2: tttctgtatcttagcgttttccg			
R: caacatcctccatagccattcca			
**Pid4-SN/CO^{T1841A}**	57	*Hph* I (37)	195/161
F: catcaatagaagcatgattcaacc			
R: tcctgtctcccatcaatagaaag			
**Pid4-SN/CO^{C2250G}**	56	*Apa* I (25)	120/92
F1: gtcacttcgaatttactacccgtgg			
F2: gatactccgatttcaatatctatgg			
dR: ggaactcygccaccaccwscga			
**Set IV. Transformation test**			
***Pid2* cloning**	62	*Asc* I (37)	6,259
F: attggcgcgccgcatcaacatagacgtagcgtgg			
R: attggcgcgccctagttacagatcactgtgccat			
***Pid3* cloning**	62	*Asc* I (37)	6,237
F: attggcgcgcccacacattgtacacctacgaccac			
R: attggcgcgccgaacgacaagtgcgacatgattg			
**HYG**	58	*n*/*a*	879
F: cttctgcgggcgatttgt			
R: cagcgtctccgacctgat			

**Vector_F/Pid2_R**	56	*n/a*	4,738
F: aattaattcctaggccaccatgttg			
R: ctatgatagccaaagttacgcg			
**Pid2_F/Vector_R**	56	*n/a*	2,114
F: ggaatacaactcgtttcgcatct			
R: gtctggaccgatggctgtgtag			
**Vector_F/Pid3_R**	60	*n/a*	4,209
F: aattaattcctaggccaccatgttg			
R: aatgacagaaggcgtccaatgtgc			
**Pid3_F/Vector_R**	60	*n/a*	2,919
F: aagagtttcgcaagaatgatcgg			
R: gtctggaccgatggctgtgtag			

[a] F, forward; R, reverse; PCR with multiple primers was designed with F1, F2, or R1, R2, if any; and degenerate primer sequences were labelled dF and dR, where r=a/g; w=a/t; k=g/t; s=c/g; and y=c/t. For transformation test, sequence unlined was responding to the common restriction enzyme *Asc* I for ligating fragments (resistance genes) into the binary vectors, pYLTAC380H.

[b] Each PCR was initiated by a 94°C/3 min denaturation, followed by 35 cycles of 94°C/30 s, 50-62°C/30 s (temperature dependent on choice of primers), 72°C/25-30 s (time dependent on amplicon size) and a final extension step of 72°C/5 min. Amplicons were electrophoresed through a 10-12% polyacrylamide gel. For transformation test, each PCR was initiated by a 95°C/3 min denaturation, followed by 35 cycles of 95°C/15 s, 50-62°C/15 s, 72°C/2-8 min and a final extension step of 72°C/5 min. Amplicons were electrophoresed through a 1% agarose gel.

[c] The number shown in parentheses refers to the temperature at which the restriction digestion reaction was run.

[d] Only the critical bands were sized.
